# MtCAS31 Aids Symbiotic Nitrogen Fixation by Protecting the Leghemoglobin MtLb120-1 Under Drought Stress in *Medicago truncatula*

**DOI:** 10.3389/fpls.2018.00633

**Published:** 2018-05-14

**Authors:** Xin Li, Hao Feng, JiangQi Wen, Jiangli Dong, Tao Wang

**Affiliations:** ^1^State Key Laboratory of Agrobiotechnology, College of Biological Sciences, China Agricultural University, Beijing, China; ^2^Plant Biology Division, Samuel Roberts Noble Foundation, Ardmore, OK, United States

**Keywords:** dehydrins, leghemoglobin, symbiotic nitrogen fixation, drought stress, nitrogen fixation efficiency

## Abstract

Symbiotic nitrogen fixation (SNF) in legume root nodules injects millions of tons of nitrogen into agricultural lands and provides ammonia to non-legume crops under N-deficient conditions. During plant growth and development, environmental stresses, such as drought, salt, cold, and heat stress are unavoidable. This raises an interesting question as to how the legumes cope with the environmental stress along with SNF. Under drought stress, dehydrin proteins are accumulated, which function as protein protector and osmotic substances. In this study, we found that the dehydrin MtCAS31 (cold-acclimation-specific 31) functions in SNF in *Medicago truncatula* during drought stress. We found that *MtCAS31* is expressed in nodules and interacts with leghemoglobin MtLb120-1. The interaction between the two proteins protects MtLb120-1 from denaturation under thermal stress *in vivo*. Compared to wild type, *cas31* mutants display a lower nitrogenase activity, a lower ATP/ADP ratio, higher expression of nodule senescence genes and higher accumulation of amyloplasts under dehydration conditions. The results suggested that MtCAS31 protects MtLb120-1 from the damage of drought stress. We identified a new function for dehydrins in SNF under drought stress, which enriches the understanding of the molecular mechanism of dehydrins.

## Introduction

The most significant characteristic of legumes is symbiotic nitrogen fixation (SNF). Approximately 68% of all ammonia fixation is done by legumes in nodules (Peoples et al., 2009; Biswas and Gresshoff, 2014). In the early stage, nod factor (NF) released from rhizobium triggers the dedifferentiation and division of root cortical cells. At the same time, rhizobium invades the roots, which is facilitated by infection threads (IT), which are tubular structures formed by invagination of the cell wall and plasma membrane. ITs ramify and release rhizobium in cortical cells by endocytosis. The released rhizobia are surrounded by a plant membrane called the symbiosome membrane. The symbiosome membrane together with the enclosed bacteria is called a symbiosome in which SNF occurs. During SNF, atmospheric N_2_ is converted into ammonia, which is catalyzed by the nitrogenase complex (Oldroyd et al., 2011; Udvardi and Poole, 2013). In the symbiosome, the nitrogenase complex is anaerobic and sensitive to oxygen (Ott et al., 2005), which makes SNF a microaerophilic process. However, a vast amount of oxygen is required for respiration and energy metabolism of the nodules and plants. Legumes have evolved three strategies to resolve this contradiction between SNF and plant growth: a gaseous barrier that inhibits oxygen influx into the central infected tissue (Minchin, 1997); a high respiration rates of bacteroides and quick oxygen consumption; and leghemoglobin, a hemoglobin in legumes that rapidly binds and releases oxygen, delivering the oxygen to the mitochondria and bacteroides of infected cells in nodules to maintain a relatively low and stable concentration of oxygen (Minchin, 1997; Arredondo-Peter et al., 1998). Through these three strategies, legumes ensure that SNF, symbiosome development and plant development occur smoothly. In mature nodules, the most abundant protein is leghemoglobin, which plays a critical role in SNF. Knockdown of leghemoglobin in *Lotus* *japonicus* resulted in increased free oxygen in nodules, nitrogenase instability, and abortion of SNF (Ott et al., 2005, 2009).

Similar to other biological processes, SNF is extremely sensitive to abiotic stresses, such as drought and high salinity; however, the mechanism underlying this response is not fully understood. The primary effect of drought stress on SNF is to promote the nodule senescence and reduce the number of nodules. Prudent et al. (2016) demonstrated that SNF is totally inhibited by drought stress and is restored following recovery from drought. The number of nodules which initiated during water recovery depends on the nitrogen nutritional index of the plant at the end of the drought period (Prudent et al., 2016). At the biochemical level, drought stress reduces the leghemoglobin content and nitrogenase activity and increases cysteine protease activity in nodules. On the other hand, Hunt and Layzell (1993) demonstrated that O_2_ limitation in symbiotic nodules was responsible for the inhibition of nitrogen fixation (Gogorcena et al., 1995; Witty and Minchin, 1998; Naya et al., 2007). Moreover, plant leaves accumulated more ureides and certain amino acids under drought, thereby indicating that the N signal may play an important role in SNF limitation under drought stress (King and Purcell, 2005; Ladrera et al., 2007; Sulieman et al., 2010). In legumes, ammonia assimilation also requires carbon compounds. However, drought stress elicits inhibition of sucrose synthase activity, which causes the accumulation of sucrose and a decrease in malate–the primary C source transported into the symbiosome (Gonzalez et al., 1995, 1998; Gordon et al., 1997; Ramos et al., 1999). Therefore, C limitation is also a factor that inhibits SNF under drought stress. Drought causes a reduction in SNF in order to rescue the productivity of legumes. Notably, under drought stress, carbohydrates are preferentially allocated to roots and leaves, thus reducing SNF to ensure survival under harmful environmental conditions. However, nodulating plants, which have higher N per leaf area, possess higher water use efficiency than that of non-nodulating plants (Adams et al., 2016). Nodulated *M. truncatula* plants recover from drought stress much more easily than do non-nodulated *M. truncatula* (Staudinger et al., 2016). Lower SNF levels are still necessary and directly affect the production of legume plants under water-limited conditions. Therefore, maintaining SNF under drought stress is an important strategy to improve the productivity of legumes. Because of the importance of leghemoglobin during SNF, maintaining the integrity and activity of leghemoglobin under drought stress is necessary to improve SNF efficiency and drought tolerance.

Dehydrins, belonging to the family of late embryogenesis abundant (LEA) proteins, are widely distributed in several plant species. Dehydrins are highly induced and are seen as key regulators under abiotic stress. Classical dehydrins contain high amounts of Gly and Lys, which lead to the hydrophilic and hydrophobic characteristics of dehydrins and an unstable secondary structure (Kovacs et al., 2008). In plant cells, dehydrins easily bind with biomolecules, such as DNA, RNA, proteins, ions, and membrane components (Hernandez-Sanchez et al., 2014). After binding with these biomolecules, dehydrins gain a stable secondary structure (Rorat, 2006). According to previous studies, dehydrins protect their target proteins from degradation under abiotic stress (Kovacs et al., 2008). However, studies regarding the molecular mechanism of dehydrins in drought response have primarily been conducted *in vitro* (Cuevas-Velazquez et al., 2014). The *in vivo* mechanism of dehydrins in drought response was first characterized in 2012 (Xie et al., 2012). Heterologous expression of the *Medicago truncatula* dehydrin MtCAS31 in *Arabidopsis* decreased stomatal density and improved drought tolerance of the plant. MtCAS31 influences stomatal development in *Arabidopsis* by interacting with a key regulator of stomatal development, AtICE1 (Xie et al., 2012). However, the other function of dehydrins, such as their role in SNF, has not been studied.

In this study, we determined a new function of dehydrin in SNF under drought stress. MtCAS31 protects leghemoglobin, MtLb120-1, by protein–protein interaction to maintain nitrogenase activity under drought stress. Meanwhile, MtCAS31 delays drought-induced nodule senescence. In conclusion, MtCAS31 reduces the negative effects of drought stress on SNF. Our study identified a new function of dehydrin in SNF in *M. truncatula* under drought stress and hence, enriches the understanding of dehydrins in drought response.

## Materials and Methods

### Plant Materials

The plant materials used in this study were *Medicago truncatula* R108 wild type, *MtCAS31* knockout mutants generated by transcription activator-like effector nuclease (TALEN) and *MtCAS31* re-transposon *Tnt1*-insertion mutant, NF5714. Three independent knockout mutants line, *cas31-27* (40 bp deletion), *cas31-34* (25 bp deletion), and *cas31-51* (8 bp deletion) were used in this study. In NF5714, *Tnt1* was inserted into the third exon of *MtCAS31*. MtCAS31 cannot be detected in protein level in the mutants.

### Design, Assembly, and Identification of Transcription Activator-Like Effector Nuclease (TALEN) Constructs of MtCAS31

Target sites are required to start with a T nucleotide for each sequence. We designed the target sequence using TAL Effector Nucleotide Targeter 2.0^1^ and used the method described by Ma et al. (2015). Each gene had a pair of target sequences. The two target sequences of *MtCAS31* had 17 and 20 repeat variable diresidues (RVDs). There was a spacer sequence between the two target sequences. The RVDs were assembled using the three-step golden gate method (Cermak et al., 2011) and then assembled into pCAMBIA1300 to obtain the final vector. *cas31*-TALEN transgenic plants were generated by the leaf-disk transformation mediated by *Agrobacterium*. TALEN knockout transgenic plants were identified by sequencing. The amplified target sequence was sequenced to identify the mutation pattern.

For genetic transformation, seeds of wild-type plants were preprocessed with 98% H_2_SO_4_ for 8 min and sterilized with 5% NaClO for 15 min. The seeds were germinated on 0.8% agar plates at room temperature. Plants were grown in chambers at 24°C under 16 h light (light intensity is 200 μmol m^-2^ s^-1^)/8 h dark conditions and 70% relative air humidity. Leaves of 4-week-old plants were detached and sterilized with 5% NaClO. The sterilized leaves were cut into square sections, soaked in *Agrobacterium* EHA105 carrying the target vectors *cas31*-TALEN, and maintained in vacuum for 20 min. After cultivation for 90 min, the leaves were placed on SH3α medium [1 mM MgSO_4_⋅7H_2_O, 1 mM KNO_3_, 1 mM (NH_4_)_2_SO_4_, 0.25 mM CaCl_2_⋅2H2O, 0.5 mM KH_2_PO_4_, 0.1 mg/L MnSO_4_⋅H_2_O, 1 mg/L H_3_BO_3_, 0.1 mg/L ZnSO_4_⋅7H_2_O, 0.1 mg/L KI, 0.01 mg/L Na_2_MoO_4_⋅2H_2_O, 0.02 mg/L CuSO_4_⋅5H_2_O, 0.01 mg/L CoCl_2_⋅6H_2_O, 0.5 mg/L nicotinic acid, 0.5 mg/L thiamine HCl, 0.5 mg/L pyridoxine HCl, 0.1 mM NaFe⋅EDTA, 4 mg/mL 2,4-D, and 0.5 mg/mL 6-BA] and cultivated for 3 weeks in the dark. The leaves dedifferentiated into calli. After being subcultured three times in SH3α, the calli were transferred to SH9 medium [(NH_4_)_2_SO_4_, 0.25 mM CaCl_2_⋅2H2O, 0.5 mM KH_2_PO_4_, 0.1 mg/L MnSO_4_⋅H_2_O, 1 mg/L H_3_BO_3_, 0.1 mg/L ZnSO_4_⋅7H_2_O, 0.1 mg/L KI, 0.01 mg/L Na_2_MoO_4_⋅2H_2_O, 0.02 mg/L CuSO_4_⋅5H_2_O, 0.01 mg/L CoCl_2_⋅6H_2_O, 0.5 mg/L nicotinic acid, 0.5 mg/L thiamine HCl, 0.5 mg/L pyridoxine HCl, and 0.1 mM NaFe⋅EDTA], cultivated under light until they differentiated into seedlings, and then transferred to 1/2 MS medium to generate roots.

### Histochemical GUS Staining

Different tissues of transgenic plants were collected and stained at 37°C in staining buffer (50 mM phosphate buffer, pH 7.0, 1 mM X-Gluc, 5 mM potassium ferricyanide, and 5 mM potassium ferrocyanide) for 16 h (Kim et al., 2002). To remove surface dye and chlorophyll after GUS staining, plant tissues were soaked in 75% ethanol for several hours. Observations were conducted using a light microscope, and a camera was used to take digital images using the corresponding OLYMPUS NIS Elements D software.

### Rhizobium Inoculation

Plants (wild type and *cas31* mutants) were grown in a vermiculite/perlite (5:2, v/v) mixture stirred with Fahraeus medium (0.5 mM MgSO_4_⋅7H_2_O, 0.7 mM KH_2_PO_4_, 0.4 mM NaH_2_PO_4_⋅2H_2_O, 10 μM Fe-EDTA, 1 μg/mL MnSO_4_, 1 μg/mL CuSO_4_, 1 μg/mL ZnSO_4_, 1 μg/mL H_3_BO_3_, and 1 μg/mL Na_2_MoO_4_) at 24°C under 16 h light (light intensity is 200 μmol m^-2^s^-1^)/8 h dark conditions and 70% relative air humidity. Seven-day-old seedlings were inoculated with rhizobium *Sinorhizobium meliloti* 1021 (*S. meliloti* 1021). For inoculation, *S. meliloti* 1021 was cultured in TY medium (3 g/L yeast extraction, 5 g/L tryptone, and 6 mM CaCl_2_) until an OD_600_ of 0.6 was reached and then centrifuged for 10 min at 5,000 rpm at room temperature. The pellet was resuspended to OD 0.5 with sterilized water. The resuspended *S. meliloti* 1021 was diluted to OD 0.05, and 20 mL *S. meliloti* 1021 was inoculated into each plant.

### Inoculation and Drought Treatment

To study SNF under drought, plants were inoculated with *S. meliloti* 1021, as described above. After inoculation, the plants were exposed to drought stress treatment. For drought stress treatment, plants were exposed to drought stress for 7 days at 24°C under 16 h light (light intensity is 200 μmol m^-2^ s^-1^)/8 h dark conditions with 40% relative air humidity, and rewatered with 100 mL water, which is considered as a drought-rewater cycle. The nodule number and nitrogenase activity of inoculated plants were noted after two drought-rewater cycles. Three replications with 15 plants each were used for statistic analysis.

### GUS Staining

To identify the expression pattern of *MtLb120-1*, 1.7 bp DNA sequence before start codon ATG was inserted into pCAMBIA1381 to drive *GUS* reporter gene. *M. truncatula* expressing *MtLb120-1pro:GUS* were inoculated with *S. meliloti* 1021. Twenty-eight days post inoculation (dpi) nodules were used for GUS staining.

For GUS staining, the nodules were soaked into GUS staining solution (100 mM PBS pH 7.0, 0.5 M potassium ferricyanide, 0.5 M potassium ferrocyanide, and 50 mg/ml X-Gluc) for overnight. The stained nodules were sliced by oscillating slicer and observed by light microscope.

### Quantitative Real-Time Reverse Transcription PCR (qRT-PCR)

qRT-PCR was performed using the SYBR Master Mix reagent using the Bio-Rad Real-Time PCR System in nodules. Expression levels of *MtCAS31*, *MtLb120-1*, *MtCP2*, *MtCP3*, *MtCP4*, *MtLECRK, MtMTD1*, and *MtMTD2* were calculated by the 2^-ΔΔCt^ method and normalized to *ACTIN* gene expression (*MtACTIN*, GenBank Accession No. XM_003602497.2).

### Yeast Two-Hybrid Assay

A yeast two-hybrid assay was used to verify the interaction between MtCAS31 and MtLb120-1. *MtCAS31* was inserted into pGBKT7 and fused with the GAL4-binding domain (pGBKT7-MtCAS31). *MtLb120-1* was inserted into pGADT7 and fused with the GAL4 active domain (pGADT7-MtLb120-1). The vectors were co-transformed into the *Saccharomyces cerevisiae* AH109 strain by PEG-mediated transformation and dropped on SD medium lacking tryptophan, leucine, adenine, and histidine (SD/-Trp/-Leu/-Ade/-His). The yeast was incubated for 48 h at 30°C. A concentration of 20 mg/mL X-α-gal was used to test the expression of the *LacZ* reporter gene.

### Biomolecular Fluorescence Complementation (BiFC)

Biomolecular fluorescence complementation (BiFC) was used to study the interaction between MtLb120-1 and MtCAS31. MtLb120-1 was fused with the C-terminus of YFP (MtLb120-1-YFP^C^), while MtCAS31 was fused with the N-terminus (MtCAS31-YFP^N^). Both constructs were driven by the CaMV35S promoter. The constructs and the negative controls (MtCAS31-YFP^N^/YFP^C^ and YFP^N^/MtLb120-1-YFP^C^) were co-transformed into *Arabidopsis* protoplasts by PEG-mediated transformation. Following a 16-h incubation, fluorescence was observed using confocal laser scanning microscopy with 488 nm excitation.

### Protein Purification and GST Pull-Down

For protein purification, *MtCAS31* was inserted in the pET30a vector and fused with the His protein tag (MtCAS31-His). *MtLb120-1* was inserted in to pGEX4T-1 and fused with the GST protein tag (GST-MtLb120-1). The recombinant protein (MtCAS31-His, GST-MtLb120-1) was purified by affinity chromatography from *E. coli.* For MtCAS31-His purification, the BL21 strains containing MtCAS31-His was used for ultrasonic decomposition and affinity chromatography by Ni^+^ affinity column. The elution protein was used for analysis.

For protein pull-down experiments, GST protein and GST-MtLb120-1 were immobilized with glutathione beads and incubated with MtCAS31-His, respectively, in binding buffer (20 mM Tris–HCl, pH 7.5, 150 mM NaCl, 3 mM MgCl_2_, 1 mM DTT, and 0.1% Triton X-100) (Xu et al., 2013) at 4°C for 3 h. The beads were then washed three times with binding buffer. The samples were boiled, separated by SDS–PAGE, and analyzed by immunoblotting assay with anti-His and anti-GST antibodies.

### Acetylene Reduction Assay

In legume nodules, nitrogenase reduces acetylene into ethylene during SNF. Therefore, the acetylene reduction assay was used to measure nitrogenase activity of wild-type plants and *cas31* mutants. Calcium carbide was added to water to generate acetylene in a Kipp’s apparatus. Nodules of wild type and *cas31* mutant plants were placed in 10-mL rubber-capped tubes. Next, 200 μL of acetylene was injected into the rubber-capped tubes and reacted for 3 h at room temperature. Following the reaction, 100 μL of the reaction gas was used for gas chromatography analysis. The peak area of ethylene was used to calculate nitrogenase activity, and the nodule weight was measured. Ethylene generated per unit nodule weight per unit time represented the activity of nitrogenase. Three replications with 15 plants each were used for statistic analysis.

### Hairy Root Transformation

The constructs that were needed for hairy root transformation (*MtLb120-1pro:GUS*, *MtLb120-1*-RNAi, *GUS*-RNAi in this study) were introduced into the *Agrobacterium rhizogenes* ARqua1 strain which is used for hairy root transformation as described and modified by Boisson-Dernier et al. (2001). In brief, the root tips of germinated seedlings were cut, and the wounds were wrapped with ARqua1 that contained the target construct. The treated seedlings were cultured in FN medium (Fahraeus medium with N) (0.5 mM MgSO_4_⋅7H_2_O, 0.7 mM KH_2_PO_4_, 0.4 mM NaH_2_PO_4_⋅2H_2_O, 1 mM NH_4_NO_3_, 10 μM Fe-EDTA, 1 μg/mL MnSO_4_, 1 μg/mL CuSO_4_, 1 μg/mL ZnSO_4_, 1 μg/mL H_3_BO_3_, and 1 μg/mL Na_2_MoO_4_) at 20°C for 7 days and then cultured at 24°C for 14 days. For rhizobium inoculation, the transformed plants were moved onto Fahraeus medium for nitrogen starvation for 7 days and then inoculated with the rhizobium *S. meliloti* 1021.

### Measurement of the ATP/ADP Ratio

Nodules of different plant materials were flash-frozen in liquid nitrogen and ground into powder in 2-mL centrifuge tubes. Perchloric acid (0.6 M) was added to the centrifuge tubes, which were vortexed for 1 min and then centrifuged at 3,000 rpm/min for 10 min at 4°C. The pH of the supernatant was adjusted to 6.5 with KOH and stabilized on ice until the perchlorate precipitated. Next, the tubes were centrifuged at 4°C for 5 min at 8,000 ×*g*. The supernatant was filtered using 0.45-μm Millipore filters before analysis by HPLC. For HPLC analysis, C18 chromatographic column (5 μm, 250 mm × 4.6 mm) were used to analyze the ADP and ATP. The mobile phase was phosphate buffer (20 mM KH_2_PO_4_, 20 mM K_2_HPO_4_, pH 7.5). Flow rate was 1.2 mL/min and the column temperature was 30°C. The measurement of light absorption is in the UV region at 254 nm. The retention time of ADP and ATP were 6.25 and 5.36 min, respectively. The peak area represents the content of ATP and ADP. Three replications with 15 plants each were used for statistics analysis.

### Thermal Inactivation Assay

To test whether MtCAS31 protects MtLb120-1, a thermal inactivation assay was performed. The method described by Kovacs et al. (2008) with modifications was used. One milligram MtCAS31-His and GST-MtLb120-1 were incubated at 75°C for 45 min in binding buffer (20 mM Tris–HCl, pH 7.5, 150 mM NaCl, 3 mM MgCl2, 1 mM DTT, and 0.1% Triton X-100). The incubated proteins were divided into two groups, one for thermal inactivation, whereas the other group was control which was not subjected to thermal activation. Following thermal reaction, proteins were analyzed by native PAGE analysis.

### Statistical Analysis

SPSS software was used for statistical analysis. Values are presented as the mean ± SD. Three replications with 15 plants each were used for statistic analysis. Comparison of multiple groups was performed using ANOVA followed by LSD *post hoc* testing to determine statistical significance. Student’s *t*-test was used to determine differences when making single comparisons.

### Accession Number

The sequence data from this article have been deposited in the NCBI database. The Accession Numbers for the genes described in this article can be found in Supplementary Table online.

### Primers

The primers for the vectors construction in this article can be found in Supplementary Data Sheet 2.

## Results

### *MtCAS31* Was Expressed in Nodules

**FIGURE 1:**
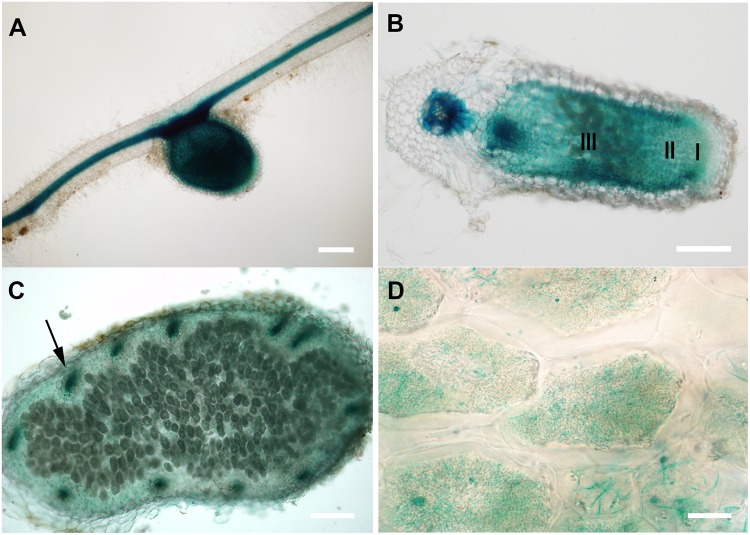
GUS staining of transgenic *M. truncatula* expressing *MtCAS31pro:GUS*. **(A)** Nodule and root; bar = 200 μm. **(B)** Longitudinal section of nodules, I represents the meristematic zone, II represents the infection zone, III represents the nitrogen fixation zone; bar = 200 μm. **(C)** Cross section of the nitrogen fixation zone of nodule; bar = 100 μm. Arrow represents the vascular bundle in nodules. **(D)** Magnified nodule cells in **C**; bar = 10 μm.

MtCAS31 is a Y_2_K_4_ dehydrin in *M. truncatula*. According to our previous study, heterologous expression of *MtCAS31pro:GUS* in *Arabidopsis thaliana* and *Nicotiana benthamiana* revealed that *MtCAS31* was expressed in the stomata and vascular tissues of roots and leaves (Xie et al., 2012). To characterize the expression patterns of *MtCAS31* in *M. truncatula*, we generated transgenic *M. truncatula* plants expressing the *MtCAS31pro:GUS* construct by *Agrobacterium* mediated transformation. The transgenic plants were inoculated with rhizobium *Sinorhizobium meliloti* strain 1021 (*S. meliloti* 1021) and were used for GUS staining at 28 dpi. Following GUS staining, the expression of *MtCAS31* was detected in nodules as well as in roots (**Figure 1A**). The longitudinal section of nodules showed that *MtCAS31* expression was detected in meristematic zone (I), infection zone (II), and nitrogen-fixation zone (III) (**Figure 1B**). The cross-section of nodules showed that *MtCAS31* was also detected in the vascular tissue of nodules (indicated by arrow) (**Figure 1C**). At higher magnifications, the expression of *MtCAS31* in nodules cells was detected (**Figure 1D**). In addition, *M. truncatula* nodule expression data^2^ revealed that the highest expression level of *MtCAS31* was the nitrogen fixation zone (Supplementary Figure S1; Roux et al., 2014). Promoter analysis of *MtCAS31* by PlantCARE^3^ showed that three *cis* elements related to nodulation were detected in *MtCAS31* promoter region (Supplementary Data Sheet 1). All these data implied that *MtCAS31* expresses in nodules. Dehydrins were well studied but the nodule-expression profile of dehydrins has not been reported until now. The results gave us a clue that dehydrin MtCAS31 may participate in SNF in nodules of *Medicago truncatula.*

### Nitrogenase Activity Is Significantly Lower in *cas31* Mutants Than in Wild-Type Plants Under Drought Stress

**FIGURE 2:**
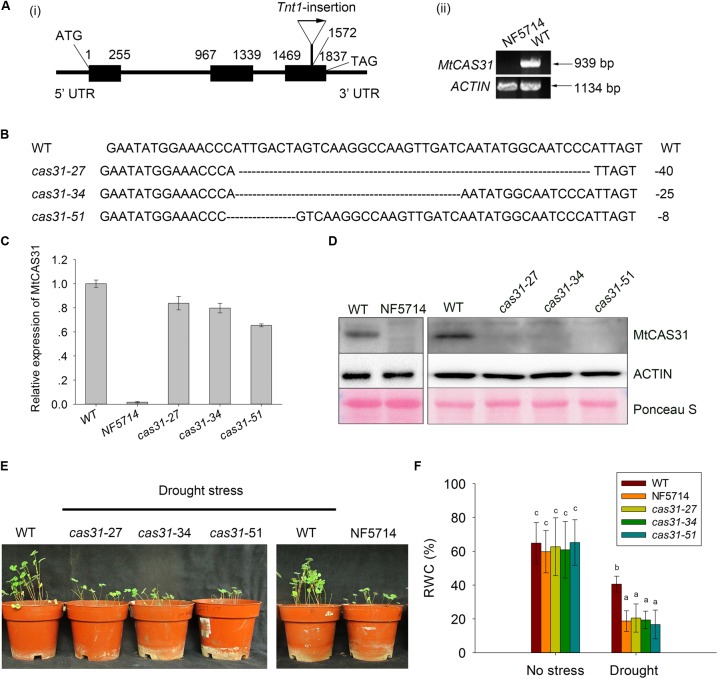
Identification of *cas31* mutants. **(A)** Identification of the retrotransposon *Tnt1*-insertion mutant, NF5714. *Tnt1*-insertion was localized in the third exon of *MtCAS31* **(i)** and the full length of *MtCAS31* cannot be detected by RT-PCR **(ii)** *ACTIN* was reference. **(B)** Identification of the *cas31* mutants generated by transcription activator-like effector nuclease (TALEN) technology. Deletions were 40, 25, 8 bp in the *cas31*-27, *cas31*-34, and *cas31*-51 knockout mutants, respectively. **(C)** Relative expression of *MtCAS31* in *cas31* mutant by qRT-PCR. Values were normalized to *ACTIN* expression. **(D)** Immunoblotting analysis of WT and *cas31* mutants with anti-MtCAS31 antibody. ACTIN was reference. **(E)** Phenotype of WT and *cas31* mutants under drought stress. **(F)** Relative water content (RWC) in WT and *cas31* mutant under drought stress. Data represent the mean ± SD (*n* = 30). Significance is indicated by letters; one-way ANOVA, LSD.

To determine the function of MtCAS31 in SNF, a retrotransposon *Tnt1*-insertion mutant (NF5714) was screened out from *Tnt1*-insertion mutant library of Samuel Roberts Noble Foundation^4^ (d’Erfurth et al., 2003; Cheng et al., 2011; Pislariu et al., 2012). The *Tnt1* insertion was localized in the third exon of *MtCAS31* in NF5714 (**Figure 2Ai**) and the full length of *MtCAS31* cannot be detected by RT-PCR (**Figure 2Aii**), suggesting that NF5714 was a null mutant. In addition, we generated *MtCAS31* knockout mutants (*cas31*-27, *cas31*-34, and *cas31*-51) using TALEN technology. The target sequence of *cas31*-knockout was designed by TAL Effector Nucleotide Targeter 2.0 (see footnote 1). A pair of target sequences was needed to recognize the genomic sequence. The two target sequences of *MtCAS31* had 17 and 20 RVDs, respectively, which were assembled using the three-step golden gate. The *cas31*-TALEN transgenic plants were generated by the leaf-disk transformation mediated by *Agrobacterium* (Duan et al., 2017) and the *cas31*-TALEN were identified by sequencing. *cas31*-27, *cas31*-34, and *cas31*-51 knockout mutants contain 40, 25, 8 bp deletion, respectively (**Figure 2B**) and lead to early termination (Supplementary Figure S5). MtCAS31 couldn’t be detected both at transcript level by RT-qPCR (The primers used in RT-qPCR were designed in the third exon) and protein level by immunoblotting analysis in the *Tnt1* mutant NF5714 (**Figures 2C,D**). In *cas31*-TALEN mutants, the transcript still could be detected but MtCAS31 protein cannot be detected, suggesting that the genomic base pair deletion couldn’t affect transcript but lead to protein abortion. These results suggested that the mutants were null mutants and could be used for the functional analysis.

Since MtCAS31 plays a role in drought response in *Arabidopsis*, the *cas31* mutants above were treated with drought stress to elucidate the function of MtCAS31 in drought response in *M. truncatula*. After treatment with drought stress, the inhibition of plant growth was much more severe in *cas31* mutants (**Figure 2E**). The relative water content (RWC) was significantly lower in *cas31* than WT (**Figure 2F**), suggesting that MtCAS31 play a positive role in drought response.

**FIGURE 3:**
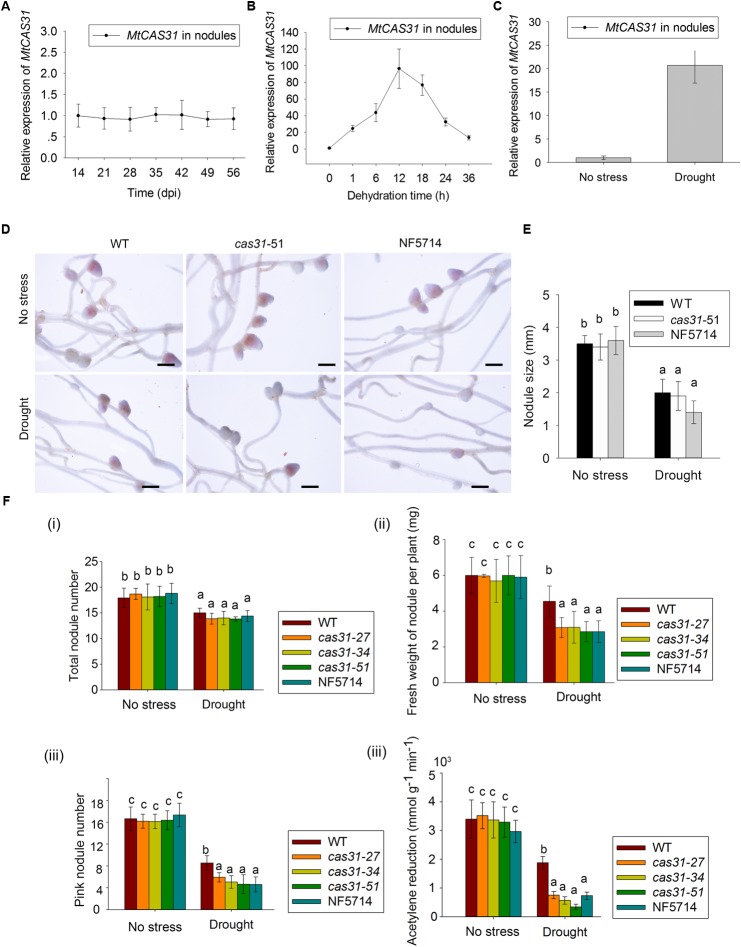
Nodules and nitrogenase activity of WT and *cas31* mutants. **(A)** Relative expression of *MtCAS31* in nodules after inoculation of rhizobium *S. meliloti* 1021 at selected time points. Values were normalized to *ACTIN* expression. Data represent the mean ± SD of three replications with 15 plants each. **(B)** Relative expression of *MtCAS31* in nodules under dehydration. Values were normalized to *ACTIN* expression. **(C)** Relative expression of *MtCAS31* in nodules under drought stress after two drought-rewater cycles (a drought-rewater cycle: withhold water for 7 days and rewater for 7 days). Values were normalized to *ACTIN* expression. **(D)** Nodules in WT and *cas31* mutant under no stress condition and drought stress; bar = 2.5 mm. **(E)** Nodule number analysis in **D**. **(F,i)** Numbers of nodule in WT and *cas31* mutants was scored at 28 dpi both under water-sufficient conditions and after two drought-rewater cycles. Data represent the mean ± SD of three replications with 15 plants each. Significance is indicated by letters; one-way ANOVA, LSD. **(ii)** Fresh weight of nodules per plant under no stress and drought stress conditions. Nodules from 28 dpi in different plant materials were used for analysis. Data represent the mean ± SD of three replications with 15 plants each. Significance is indicated by letters, one-way ANOVA, LSD. **(iii)** Pink nodules under no stress and drought stress conditions. Nodules from 28 dpi in different plant materials were used for analysis. Data represent the mean ± SD of three replications with 15 plants each. Significance is indicated by letters, one-way ANOVA, LSD. **(iv)** Acetylene reduction assay was used to analyze the nitrogenase activity in WT and *cas31* mutants under no stress and drought stress conditions. Acetylene reduction represents nitrogenase activity. Seven-day-old seedlings were inoculated with rhizobium *S. meliloti* 1021. Seven days later, the inoculated plants were exposed to drought-rewater cycles. Acetylene reduction assay was conducted after two drought-rewater cycles (a drought-rewater cycle: withhold water for 7 days and rewater for 7 days) and under no stress conditions. Data represent the mean ± SD of three replications with 15 plants each. Significance is indicated by letters; one-way ANOVA, LSD.

To elucidate the role of MtCAS31 in SNF, we first analyzed the transcript level of *MtCAS31* in nodules of *M. truncatula* wild type after inoculation with *S. meliloti* 1021 at 14, 21, 28, and 35 dpi. Expression levels of *MtCAS31* measured by qRT-PCR did not show any changes in nodules at the selected time point after inoculation (**Figure 3A**), suggesting that the expression level of *MtCAS31* was not changed at different nodule developmental stage in nodules. Since dehydrins were accumulated under drought stress and function as a drought regulator, we analyzed the relative expression of *MtCAS31* in nodules under dehydration (PEG 8000, 30%, w/v). Seven days wild-type seedlings were inoculated with *S. meliloti* 1021 for 4 weeks, then treated with 30% PEG 8000 (w/v) for different time period (0, 6, 12, 18, and 24 h). The relative expression of *MtCAS31* was highly induced in nodules after dehydration (**Figure 3B**) by qRT-PCR, indicating that MtCAS31 functions in dehydration-simulated drought stress in nodules. To confirm the result, wild type was treated with drought stress after inoculation. qRT-PCR was used to analyze the relative expression of *MtCAS31* in nodule at 28 dpi. Like dehydration, drought stress could highly induce the expression of *MtCAS31* in nodule (**Figure 3C**), suggesting that *MtCAS31* functions in drought response in nodules.

To analyze the function of MtCAS31 in drought response in nodules, wild type and *cas31* mutant were treated with drought stress after inoculation as follow: 7 days seedlings were inoculated with *S. meliloti* 1021, and the inoculated plants were exposed to drought stress. The photos of WT and *cas31* mutant (*cas31*-51 and NF5714 were shown as examples) nodules were taken at 28 dpi (**Figure 3D**). The nodule number and size showed no difference between wild type and *cas31* mutant both in well-watered (no stress) conditions and drought stress (**Figure 3E**). Under drought stress, the nodule numbers of wild type and *cas31* mutants were both reduced, which didn’t show any difference (**Figures 3D,Fi**). The fresh weight of nodule per plant was showed no difference under well-watered condition but significantly lower in *cas31* mutant than in wild type (**Figure 3Fii**), suggesting that the drought stress negatively affect the development of nodule, in which process MtCAS31 plays a critical role. Interestingly, we noticed that the number of the pink nodule was much less in *cas31* mutants than wild type under drought stress, (**Figures 3D,Fiii**), indicating that *cas31* mutants had less effective nodules than wild type under drought stress since pink nodules are considered as the effective nodules. Less effective nodules mean less SNF ability which is represented by nitrogenase activity (Oldroyd et al., 2011; Udvardi and Poole, 2013). Therefore, we analyzed nitrogenase activity in wild type and *cas31* mutants under both well-watered (no stress) and drought stress conditions by acetylene reduction assay. The result revealed that the production of ethylene was significantly lower in *cas31* mutants than that in wild type under drought stress (**Figure 3Fiv**), indicating that nitrogenase activity in *cas31* mutants was significantly lower than that in wild type under drought stress. The result suggested that MtCAS31 affected the SNF efficiency under drought stress.

### MtCAS31 Protects MtLb120-1 by Protein–Protein Interaction Under Stress Conditions

**FIGURE 4:**
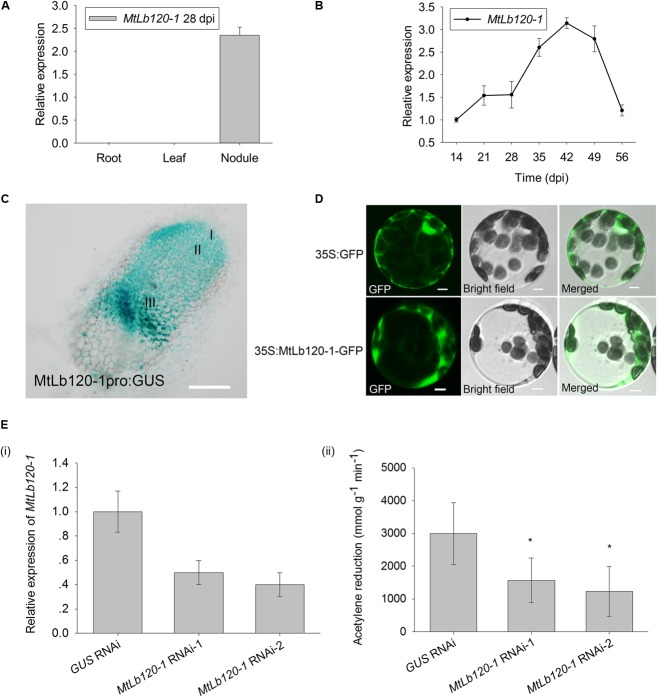
Expression pattern of *MtLb120-1*. **(A)** Relative expression of *MtLb120-1* in different tissues of *M. truncatula* at 28 dpi. Values were normalized to *ACTIN* expression. The data represent the mean ± SD of three technical replicates. **(B)** Relative expression of *MtLb120-1* in nodules at different inoculation time points. *M. truncatula* was inoculated with *S. meliloti* 1021. Nodules at different inoculation time points were used for analysis. Values were normalized to *ACTIN* expression. The data represent the mean ± SD of three technical replicates. **(C)** Histochemical GUS staining of nodules in *M. truncatula* expressing *MtLb120-1pro:GUS* by hairy root transformation; bar = 100 μm. **(D)** Subcellular localization of MtLb120-1. MtLb120-1 fused with GFP driven by the CaMV35S promoter was transformed into *Arabidopsis* protoplasts. The fluorescence was detected by confocal laser scanning microscopy at 488 nm excitation. GFP driven by the *CaMV35S* promoter is used as a control; bar = 5 μm. **(E)** Nitrogenase activity of *MtLb120-1* RNAi lines. (i) The relative expression level of *MtLb120-1* in *MtLb120-1* RNAi lines. (ii) Acetylene reduction represents nitrogenase activity *MtLb120-1* RNAi lines. *GUS* RNAi is used as the control. Data represent the mean ± SD of three replications with 15 plants each. ^∗^*P* ≤ 0.05, Student’s *t*-test.

To further investigate how MtCAS31 affects nitrogenase activity under drought stress, MtCAS31 was used as bait to screen the *M. truncatula* cDNA library to identify the interacting protein of MtCAS31. We analyzed the candidate interacting partners and identified a leghemoglobin protein, MtLb120-1. Leghemoglobin directly affects nitrogenase activity during SNF (Ott et al., 2005, 2009) and plays a critical role in SNF. SWISS^5^ prediction showed that MtLb120-1 contains 8 α-helixes (1-8 helix). Histidine in the 5th helix (His71) and the 6th helix (His94) are essential for heme prosthetic group binding (Supplementary Figure S2A). According to phylogenetic analysis, MtLb120-1 showed a close relationship with LjLb1 and LjLb2 (Gene ID: Lj5g3v0035290 and Lj3g3v3338170) and clustered far away from other leghemoglobins in *M. truncatula* such as MtLb1 (*Medicago* gene ID: Medtr5g066070), MtLb2 (*Medicago* gene ID: Medtr1g090810), MtLb3 (*Medicago* gene ID: Medtr5g081000), MtLb29 (*Medicago* gene ID: Medtr1g049330), MtLb (*Medicago* gene ID: Medtr4g068870), MtLb (*Medicago* gene ID: Medtr4g068860), MtLb (*Medicago* gene ID: Medtr5g080400), MtLb (*Medicago* gene ID: Medtr1g090820), MtLb (*Medicago* gene ID: Medtr7g110180), MtLb (*Medicago* gene ID: Medtr5g041610), MtLb (*Medicago* gene ID: Medtr0026s0210), MtLb (*Medicago* gene ID: Medtr1g011540), and MtLb (*Medicago* gene ID: Medtr5g081030) (Supplementary Figure S2B). In *Lotus japonicus*, knock down of *LjLb2* lead to the abnormal development of bacteroid and abolishment of SNF (Ott et al., 2005, 2009). To further understand the function of MtLb120-1 in *M. truncatula*, the expression pattern of *MtLb120-1* was detected by qRT-PCR. Seven days seedling was inoculated with *S. meliloti* 1021. The nodules at different inoculation time point were used for analysis. As expected, expression of *MtLb120-1* was detected in nodules but not in roots or leaves at 28 dpi (**Figure 4A**). In nodules, the expression of *MtLb120-1* increased with nodule maturation, peaked at 42 dpi, and gradually decreased with nodule senescence (49–54 dpi) (**Figure 4B**), indicating that MtLb120-1 function in the mature nodules. Histochemical staining was used to further explore the precise expression in nodules. 1.7 kb promoter of *MtLb120-1* was inserted into pCAMBIA1381 vector to drive *GUS* reporter gene (*MtLb120-1pro:GUS*). *MtLb120-1pro:GUS* was transformed into *M. truncatula* via hairy root transformation. The transgenic roots were inoculated with *S. meliloti* 1021. Nodules at 28 dpi were stained by GUS staining and observed by microscopy. The result showed that GUS activity was mainly detected in the nitrogen fixation zone (III) (**Figure 4C**), which is consistent with the function of leghemoglobin. Interestingly, the GUS activity was also detected in infection zone (II). We speculated that MtLb120-1 also affect the development of bacteroid because knock down of leghemoglobin LjLb2 of *Louts japonicus* lead to the abnormal development of bacteroid and abolishment of SNF (Ott et al., 2005, 2009). Subcellular localization of MtLb120-1 was also determined. MtLb120-1 fused with GFP was driven by *CaMV35S* promoter and expressed in *Arabidopsis* protoplasts. After 16-h incubation at 22°C, the GFP signal was detected using confocal laser scanning microscopy with 488 nm excitation. We found that the GFP signal was detected in cytoplasm (**Figure 4D**), indicating that MtLb120-1 function in the cytoplasm.

In legumes, the absence of leghemoglobin leads to the abortion of SNF. To explore the function of MtLb120-1, we generated the *MtLb120-1* knock-down plants using RNA interference technology by *Agrobacterium* medicated hairy root transformation. The relative expression of *MtLb120-1* was decreased in *MtLb120-1* RNAi lines compared to *GUS* RNAi lines (**Figure 4Ei**). To rule out the possibility that other Lbs were also knocked down in the *MtLb120-1* RNAi lines, the expression of two other leghemoglobin genes, *MtLb1* and *MtLb2*, was quantified by qRT-PCR. The relative expression of *MtLb1* and *MtLb2* did not decrease in the transgenic lines (Supplementary Figure S3). The nitrogenase activity of *MtLb120-1* RNAi lines were analyzed at 28 dpi by acetylene reduction assay. The result showed that the nitrogenase activity of *MtLb120-1* RNAi lines was significantly lower than *GUS* RNAi (**Figure 4Eii**), indicating that MtLb120-1 influences the nitrogenase activity and functions as a major leghemoglobin in *M. truncatula*.

**FIGURE 5:**
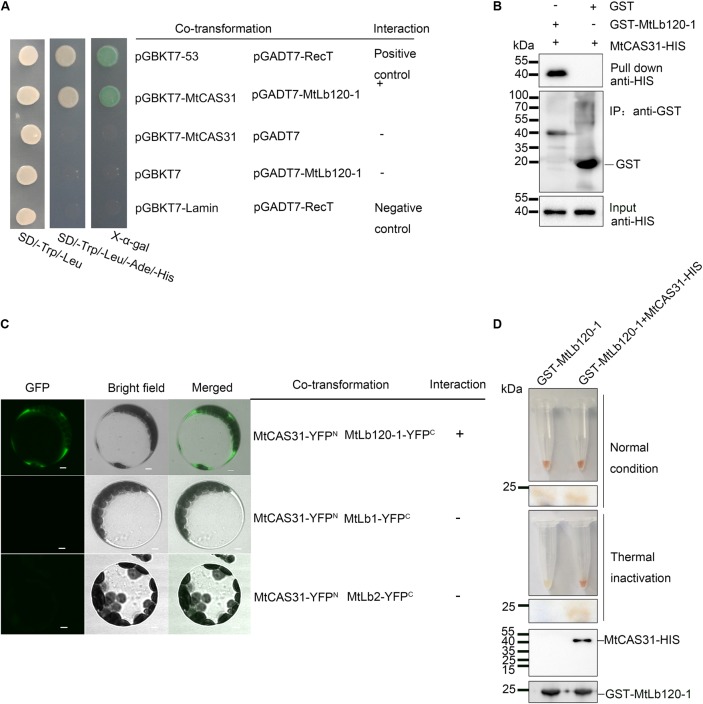
Interaction between MtCAS31 and MtLb120-1. **(A)** Yeast two-hybrid assay to determine the interaction between MtCAS31 and MtLb120-1. *MtCAS31* was inserted into pGBKT7 and fused with BD. MtLb120-1 was inserted into pGADT7 and fused with AD. pGBKT7-53/pGADT7-RecT was the positive control. pGBKT7-MtCAS31/pGADT7, pGBKT7/pGADT7-MtLb120-1, and pGBKT7-Lamin/pGADT7-RecT were negative controls. Different co-transformed AH109 yeast cells were dropped on synthetic dropout medium (SD/-Trp/-Leu/-Ade/-His) with 20 mg/mL X-α-gal. **(B)** *In vitro* GST pull-down between MtLb120-1 and MtCAS31. Recombinant proteins GST-MtLb120-1 and MtCAS31-His were purified from *E. coli*. GST-MtLb120-1 was incubated with glutathione beads and incubated with MtCAS31-His. Proteins were eluted from the beads and immunoblotted with anti-His antibody and anti-GST antibody. **(C)** Interaction between MtCAS31 and MtLb120-1 analyzed by bimolecular fluorescence complementation (BiFC). MtCAS31 was fused with the N-terminus of YFP (MtCAS31-YFP^N^). MtLb120-1 was fused with the C-terminus of YFP (MtLb120-1-YFP^C^). *Arabidopsis* protoplasts were co-transformed with the indicated constructs and incubated for 16 h. MtCAS31-YFP^N^/MtLb1-YFP^C^ and MtCAS31-YFP^N^/MtLb2-YFP^C^ were negative controls. Fluorescence signals were observed by confocal laser scanning microscopy with 488 nm excitation. YFP, yellow fluorescent protein; bar = 5 μm. **(D)** Thermal inactivation assay to determine the protection of MtLb120-1 by MtCAS31. MtLb120-1-GST incubated with or without MtCAS31-His for 4 h. Proteins were heated to 75°C for 45 min for thermal inactivation and then analyzed by native PAGE and immunoblotting with anti-GST and anti-His.

To further verify the interaction between MtCAS31 and MtLb120-1, yeast two-hybrid assays were performed. *MtCAS31* was inserted into the pGBKT7 vector and fused with the GAL4-binding domain (pGBKT7-MtCAS31). At the same time, *MtLb120-1* was inserted into the pGADT7 vector and fused with the GAL4 active domain (pGADT7-MtLb120-1). *S. cerevisiae* AH109 cells co-expressing pGBKT7-MtCAS31 and pGADT7-MtLb120-1 were gown in SD medium (SD/-Trp/-Leu/-Ade/-His) and *LacZ* reporter gene was activated, indicating that MtCAS31 interacted with MtLb120-1 in yeast cells (**Figure 5A**). GST pull-down assays were also performed to evaluate the interaction. *MtCAS31* inserted into pET30a fused with the His protein tag (MtCAS31-His), and *MtLb120-1* inserted into pGEX4T-1 fused with the GST protein tag (GST-MtLb120-1). MtCAS31-His and GST-MtLb120-1 recombinant proteins were prepared by affinity chromatography. MtCAS31-His and GST-MtLb120-1 recombinant proteins were incubated with glutathione sepharose for 4 h. Then the proteins were eluted and analyzed by anti-His antibody. The results showed that MtCAS31-His was detected in the precipitates of GST-MtLb120-1, indicating that MtLb120-1 and MtCAS31 interacted with each other (**Figure 5B**). Moreover, bimolecular fluorescence complementation (BiFC) assays were employed to examine the interaction between MtCAS31 and MtLb120-1 *in vivo*. MtLb120-1 fused with the C-terminus of YFP (MtLb120-1-YFP^C^) and MtCAS31 fused with the N-terminus of YFP (MtCAS31-YFP^N^). MtLb120-1-YFP^C^ and MtCAS31-YFP^N^ were transiently co-expressed in *Arabidopsis* protoplasts. The fluorescence signal was detected by confocal laser scanning microscopy with 488 nm excitation. The result showed that the fluorescence signal was detected in the cytoplasm after co-transformation with MtLb120-1-YFP^C^ and MtCAS31-YFP^N^. When co-transformed MtCAS31-YFP^N^ with MtLb1-YFP^C^ or MtLb2-YFP^C^, which were seen as negative control, the fluorescence signal cannot be detected (**Figure 5C**). These results suggest that MtCAS31 specifically interacts with MtLb120-1 in the cytoplasm. We speculated that the reason why MtCAS31 interacted with MtLb120-1 rather than with other leghemoglobins may be differences in structure, which are suggested by the evolutionary distance to other leghemoglobins (Supplementary Figure S2B).

Dehydrins are known as protein protector under stress conditions (Hara et al., 2005; Hara, 2010; Cuevas-Velazquez et al., 2014; Graether and Boddington, 2014). Therefore, we presumed that MtCAS31 protects MtLb120-1 through protein–protein interaction under stress. Leghemoglobin exhibits a red color under normal conditions because of the oxygen-binding ferroheme. However, the color changes due to the protein denaturation under stress conditions (Navascués et al., 2011). Since MtCAS31 and MtLb120-1 protein were both purified from *E. coli* and there is no proper method to measure the protein protection with recombinant protein *in vitro* under drought stress, we performed a thermal inactivation assay to test the protective effect of MtCAS31 on MtLb120-1 *in vitro* as described previously (Kovacs et al., 2008). MtCAS31-His and GST-MtLb120-1 recombinant proteins used in the GST pull-down were used for the thermal inactivation. 1 mg GST-MtLb120-1 was incubated with or without MtCAS31-His in binding buffer for 3 h. Then the mixture was exposed to 75°C for 45 min for thermal inactivation. After thermal inactivation, native PAGE without SDS was used for analysis. The results showed that MtLb120-1 was colorless without the presence of MtCAS31 after thermal inactivation. However, MtLb120-1 was still red with the existence of MtCAS31 (**Figure 5D**), indicating that MtCAS31 protects MtLb120-1 from denaturation under stress conditions.

### MtCAS31 Delays Drought-Induced Nodule Senescence

**FIGURE 6:**
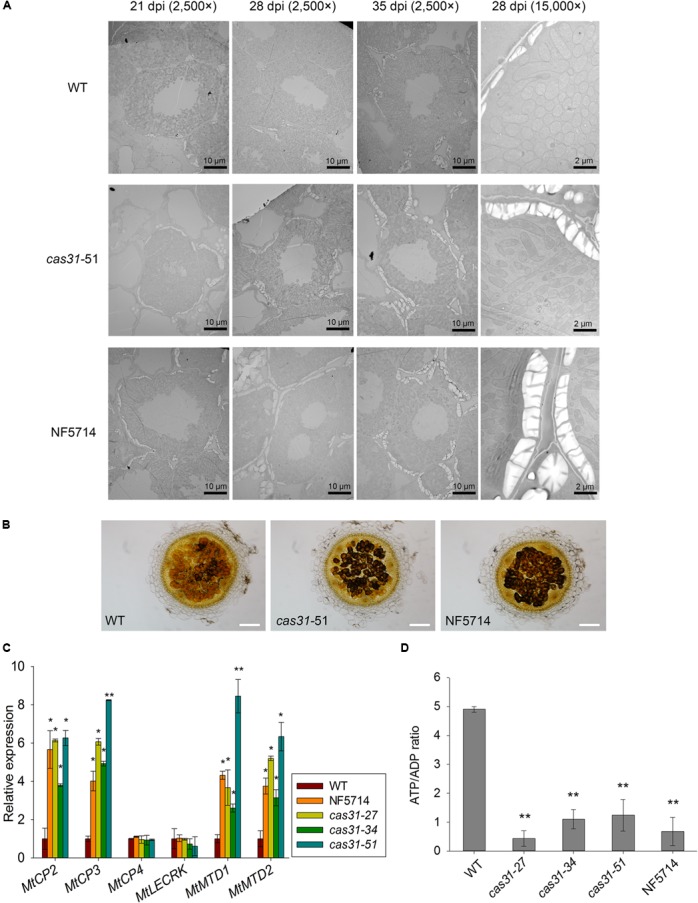
MtCAS31 delays drought-induced nodule senescence in *M. truncatula*. **(A)** Electron micrographs of nodule cross-sections of WT and *cas31* mutants under drought stress at 21, 28, and 35 dpi with 2,500× magnification. The nodules at 28 dpi were further observed at 15,000× magnification. **(B)** Cross section of 28 dpi nodules of WT and *cas31* mutants were stained by Lugol reagent; bar = 100 μm. **(C)** Relative expression of nodule senescence markers in WT and *cas31* mutants under drought stress at 28 dpi. Values were normalized to *ACTIN* expression. The data represent the mean ± SD of three replicates. **(D)** ATP/ADP ratio in nodules of WT and *cas31* mutants under drought stress determined by HPLC. Nodules at 28 dpi were used for analysis. The data represent the mean ± SD of three replications with 15 plants each. Asterisks represent statistically significant differences between WT and cas31 mutant plants, ^∗^*P* ≤ 0.1, ^∗∗^*P* ≤ 0.01, Student’s *t*-test.

Like other biological processes, SNF is negatively regulated by drought stress. To further explore the function of MtCAS31 in SNF under drought stress, electron microscopy was used to detect the differences in morphology of nodules between *cas31* mutants and wild type under drought stress. After inoculating with *S. meliloti* 1021, the wild type and *cas31* mutants (*cas31*-51 and NF5714 as examples) were exposed to drought stress. Nodules were observed at 21, 28, and 35 dpi using transmission electron microscopy. We observed that starting with 28 dpi, the nodules accumulated more amyloplasts in *cas31* mutants. With a higher magnification (15,000×), nodules at 28 dpi clearly showed more amyloplasts accumulation in *cas31* mutants (**Figure 6A**). To further verify the results, 28 dpi nodules were stained by Lugol solution, which is for amyloplasts staining. In accordance with the results of transmission electron microscopy, the staining of *cas31* mutant nodules was darker than that of wild type by light microscopy (**Figure 6B**), indicating that more amyloplasts were accumulated in *cas31* mutant nodules. The accumulation of amyloplasts indicated the altered nodule metabolism, which is observed during senescence of nodules (Puppo et al., 2005; Clement et al., 2008; de Zelicourt et al., 2012). Under drought stress, the relative expression of drought-induced senescence gene *MtCP2* and *MtCP3* (Pérez Guerra et al., 2010), *MtMTD1* and *MtMTD2* (Curioni et al., 2000) were highly induced (Supplementary Figure S4). Therefore, we analyzed these marker genes in wild type and *cas31* mutants. Relative expression of drought-induced nodule senescence marker genes such as *MtCP3*, *MtCP2*, *MtMTD1*, and *MtMTD2* were significantly higher in *cas31* nodules than in wild type after drought stress (**Figure 6C**). However, there was no difference of two developmental senescence genes, cysteine protease *MtCP4* and lectin receptor kinase *MtLECRK* (Fedorova et al., 2002; Pérez Guerra et al., 2010) between wild type and *cas31* mutants under drought stress. This result indicates that MtCAS31 retards drought-induced nodule senescence, possibly by interacting with and protecting MtLb120-1 under drought stress.

The accumulation of amyloplasts may also be caused by decreased metabolic activity. We analyzed the ratio of ATP/ADP in nodules, which reflects the metabolic level. After inoculation and treated with drought stress, the ratio ATP/ADP in *cas31* mutant nodules was significantly lower than wild type (**Figure 6D**), suggesting that the metabolic activity was significantly lower in *cas31* mutants than WT.

## Discussion

Symbiotic nitrogen fixation (SNF), which is catalyzed by nitrogenase in legume root nodules, mediates fixation of approximately 60 million tons of nitrogen into agricultural lands and supplies ammonia for non-legume crops. Similar to other biological processes, SNF is negatively affected by drought stress. Dealing with SNF and the stress response is essential for legumes, as well as for maintaining agricultural productivity. Dehydrins are accumulated under abiotic stress and work as osmotic regulation substances and protein protector, which are considered as positive regulators in abiotic stress response (Remus-Borel et al., 2010; Yamasaki et al., 2013). However, their role in SNF under stress conditions has not been studied. In this study, we identified a protein in *M. truncatula* MtLb120-1, which interacts with dehydrin MtCAS31. The interaction between the two proteins protects MtLb120-1 from denaturation under stress. The protection of MtLb120-1 under stress aids SNF under drought stress. Our study explored a new biological function of dehydrin and hence enriched the understanding of dehydrin in drought response.

Under drought stress, SNF is totally inhibited and is restored following recovery from drought. The nodule number is reduced, and earlier nodule senescence is observed (Li et al., 2009; Marquez-Garcia et al., 2015). Furthermore, the leghemoglobin content and nitrogenase activity are highly reduced. However, the regulatory mechanisms involved in this inhibition remain controversial. O_2_ limitation, C limitation, and N-signal feedback limitation are the primary hypotheses for SNF limitation under drought stress (Hunt and Layzell, 1993; Gonzalez et al., 1995; Ramos et al., 1999; Larrainzar et al., 2009). Under drought stress, the N signal plays a feedback role in SNF limitation (King and Purcell, 2005; Ladrera et al., 2007; Sulieman et al., 2010). C limitation is also a factor that inhibits SNF under drought stress. Drought stress elicits inhibition of the C source transported into the symbiosome (Gonzalez et al., 1995; Gordon et al., 1997; Ramos et al., 1999), which in turn inhibits SNF since ammonia assimilation requires carbon compounds. Moreover, drought stress causes O limitation to hamper SNF. Under drought stress, leghemoglobin was denatured or aggregated (Minchin, 1997; Figueiredo et al., 2008; Sainz et al., 2015; Mouradi et al., 2016), which led to a high oxygen concentration in nodules and the abolishment of SNF. The absence of leghemoglobin in *Lotus japonicus* results in undetectable levels of the nitrogenase protein in nodules (Ott et al., 2005, 2009). In accordance, knockdown of *MtLb120-1* via RNA interference caused reduced nitrogenase activity (**Figure 4E**). Since leghemoglobin is such an important protein, a protection mechanism to protect it from damage under environmental stress must exist; however, such a mechanism has not been reported before. We observed that MtCAS31 interacted with leghemoglobin MtLb120-1 and protected MtLb120-1 from denaturation under stress conditions (**Figure 5**). However, we believe that the protection of MtLb120-1 by MtCAS31 is not unique. Other proteins not rule out other leghemoglobin are protected by MtCAS31 in SNF; further study is required to test this hypothesis.

In the present study, we found that the RWC and plant and nodule biomasses were significantly lower than WT under drought stress indicating that *cas31* is more sensitive to drought stress. Whether this phenotype may be related to shoots and/or roots remains to be elucidated. Our hypothesis is that MtCAS31 protects MtLb120-1 to maintain the SNF activity. Indeed this may explain the lower SNF correlated to the drought sensitivity of *cas31*. However, it may be not the unique reason: because plant growth is reduced under these conditions, the whole plant N demand that pilots symbiosis is likely to be lower and therefore SNF may be indirectly reduced in cas31 as compared to the WT. In addition, drought stress causes drought-induced senescence of plants and triggers the expression of senescence-related genes (Clement et al., 2008; de Zelicourt et al., 2012). MtCAS31 protects MtLb120-1 and reduces drought-induced senescence, maintains the ATP/ADP ratio under drought stress (**Figure 6**). It is also likely that drought stress reduces the general metabolic activity of nodule cells and in turns reducing the differentiation of bacterial and nodule cells, which induces early senescence of the nodules. However, regardless of the mechanism involved, MtCAS31 aids SNF under drought stress.

In our previously study, MtCAS31 highly improved drought tolerance of transgenic *Arabidopsis.* MtCAS31 interacted with a key transcript factor of stomatal developmental AtICEI in *Arabidopsis*, which decreased the stomatal density and reduced the water loss under drought stress (Xie et al., 2012). However, the function of MtCAS31 in *M. truncatula* has not been reported. MtCAS31 was used as bait to screen *M. truncatula* cDNA library to identify MtCAS31 interacting partner. Many proteins were identified and we choose MtLb120-1 to study the function of MtCAS31 in SNF. Besides MtLb120-1, proteins involved in osmotic regulation, metallic enzyme and many other proteins were identified. We speculated that the function of MtCAS31 in drought response is multiple. MtCAS31 can reduce the negative effect on SNF in nodules under drought stress. On the other hand, MtCAS31 can regulate the drought response in non-nodule tissues by interacting with other protein through a pathway still to be defined, which will require further study.

## Conclusion

In conclusion, we demonstrated that MtCAS31 protects MtLb120-1 from denaturation under stress through protein–protein interactions and delays drought-induced nodule senescence, which in turn aids SNF under drought stress.

## Author Contributions

TW and JD designed the research. XL performed the main experiment and HF participated in the data analysis. JW provided the *Tnt1* insertion mutant. XL, JD, and TW wrote the article.

## Conflict of Interest Statement

The authors declare that the research was conducted in the absence of any commercial or financial relationships that could be construed as a potential conflict of interest.

## Footnotes

1 https://tale-nt.cac.cornell.edu/

2 https://iant.toulouse.inra.fr/symbimics

3 http://bioinformatics.psb.ugent.be/webtools/plantcare/html/

4 https://medicago-mutant.noble.org/mutant/

5 https://swissmodel.expasy.org/interactive
