# Supplementary material for: MtCAS31 Aids Symbiotic Nitrogen Fixation by Protecting the Leghemoglobin MtLb120-1 Under Drought Stress in Medicago truncatula
Source: Front Plant Sci. 2018 May 14;9:633. doi: 10.3389/fpls.2018.00633 (PMC5960688; doi:10.3389/fpls.2018.00633)
Supplement: Supplementary file 1 [file Table_1.DOCX]

**Supplementary Table.** Gene IDs of the genes used for study and phylogenetic analysis in this study

| **Sequence Name** | **Organism** | **Gene ID** |
| --- | --- | --- |
| MtCAS31 | *Medicago truncatula* | Medtr6g084640 |
| MtLb120-1 | *Medicago truncatula* | Medtr5g080440 |
| MtCP2 | *Medicago truncatula* | Medtr4g107930 |
| MtCP3 | *Medicago truncatula* | Medtr4g079470 |
| MtCP4 | *Medicago truncatula* | Medtr4g079800 |
| MtLECRK | *Medicago truncatula* | Medtr5g006160 |
| MtMTD1 | *Medicago truncatula* | Medtr1g015650 |
| MtMTD2 | *Medicago truncatula* | Medtr5g066170 |
| LjLb1 | *Lotus japonicus* | Lj5g3v0035290 |
| LjLb2 | *Lotus japonicus* | Lj3g3v3338170 |
| LjLb3 | *Lotus japonicus* | Lj5g3v0465970 |
| LjNSG2 | *Lotus japonicus* | Lj4g3v0353440 |
| MtLb1 | *Medicago truncatula* | Medtr5g066070 |
| MtLb2 | *Medicago truncatula* | Medtr1g090810 |
| MtLb3 | *Medicago truncatula* | Medtr5g081000 |
| MtLb29 | *Medicago truncatula* | Medtr1g049330 |
| MtLb | *Medicago truncatula* | Medtr4g068870 |
| MtLb | *Medicago truncatula* | Medtr4g068860 |
| MtLb | *Medicago truncatula* | Medtr5g080400 |
| MtLb | *Medicago truncatula* | Medtr1g090820 |
| MtLb | *Medicago truncatula* | Medtr7g110180 |
| MtLb | *Medicago truncatula* | Medtr5g041610 |
| MtLb | *Medicago truncatula* | Medtr0026s0210 |
| MtLb | *Medicago truncatula* | Medtr1g011540 |
| MtLb | *Medicago truncatula* | Medtr5g081030 |
