# Supplementary figures and images for: MtCAS31 Aids Symbiotic Nitrogen Fixation by Protecting the Leghemoglobin MtLb120-1 Under Drought Stress in Medicago truncatula
Source: Front Plant Sci. 2018 May 14;9:633. doi: 10.3389/fpls.2018.00633 (PMC5960688; doi:10.3389/fpls.2018.00633)

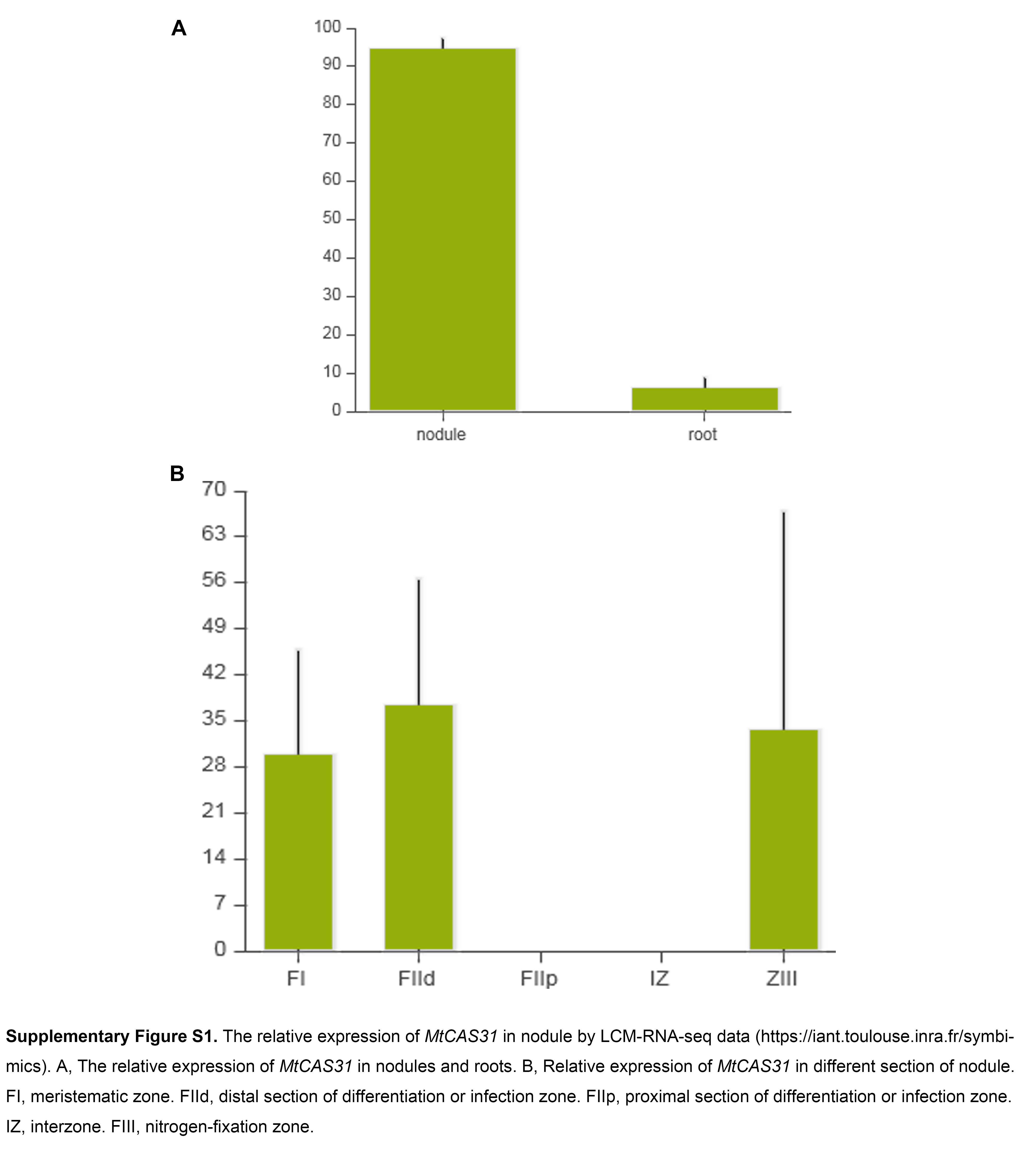

Supplement: Supplementary file 4 [file Image_1.TIF]

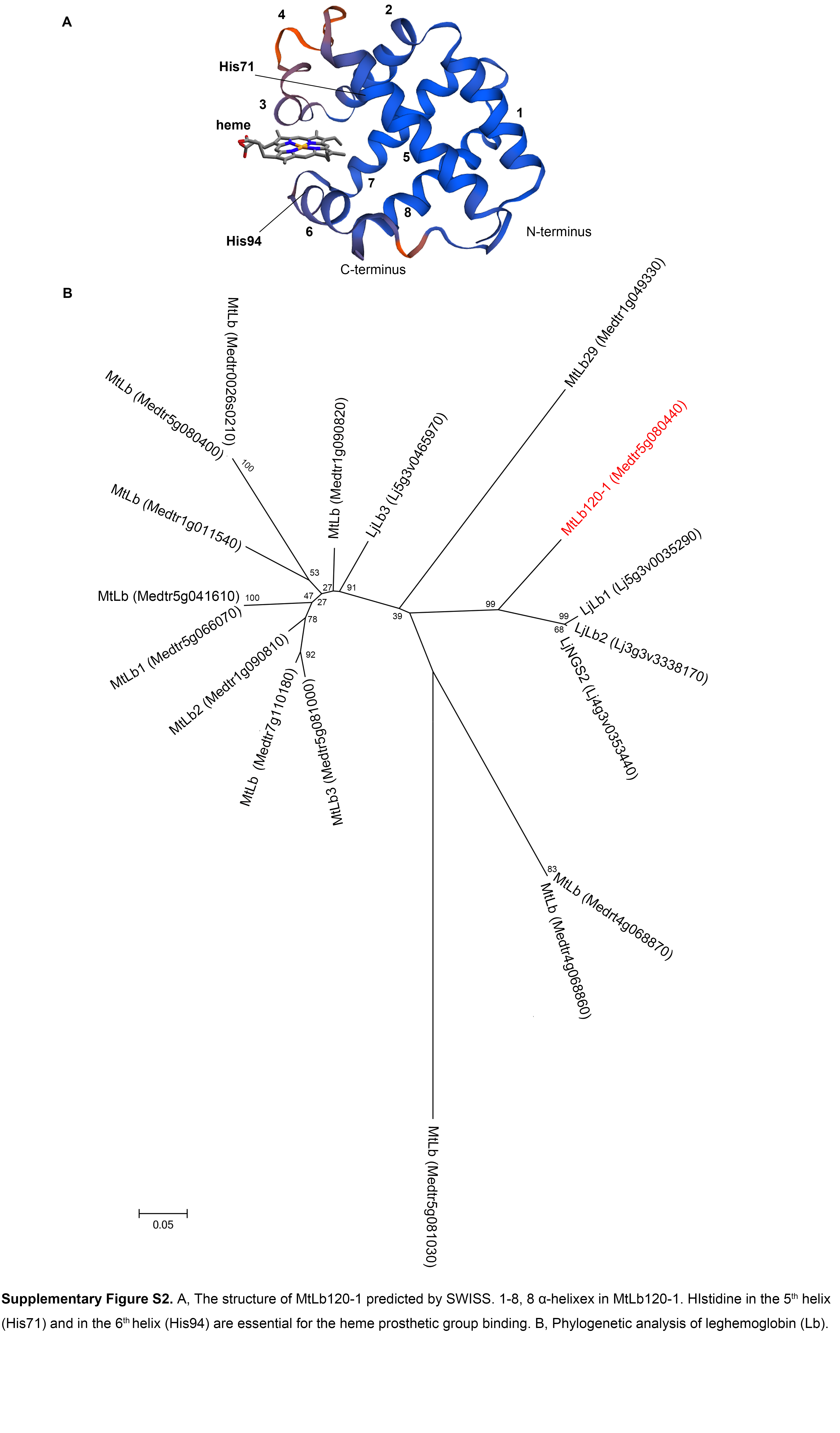

Supplement: Supplementary file 5 [file Image_2.TIF]

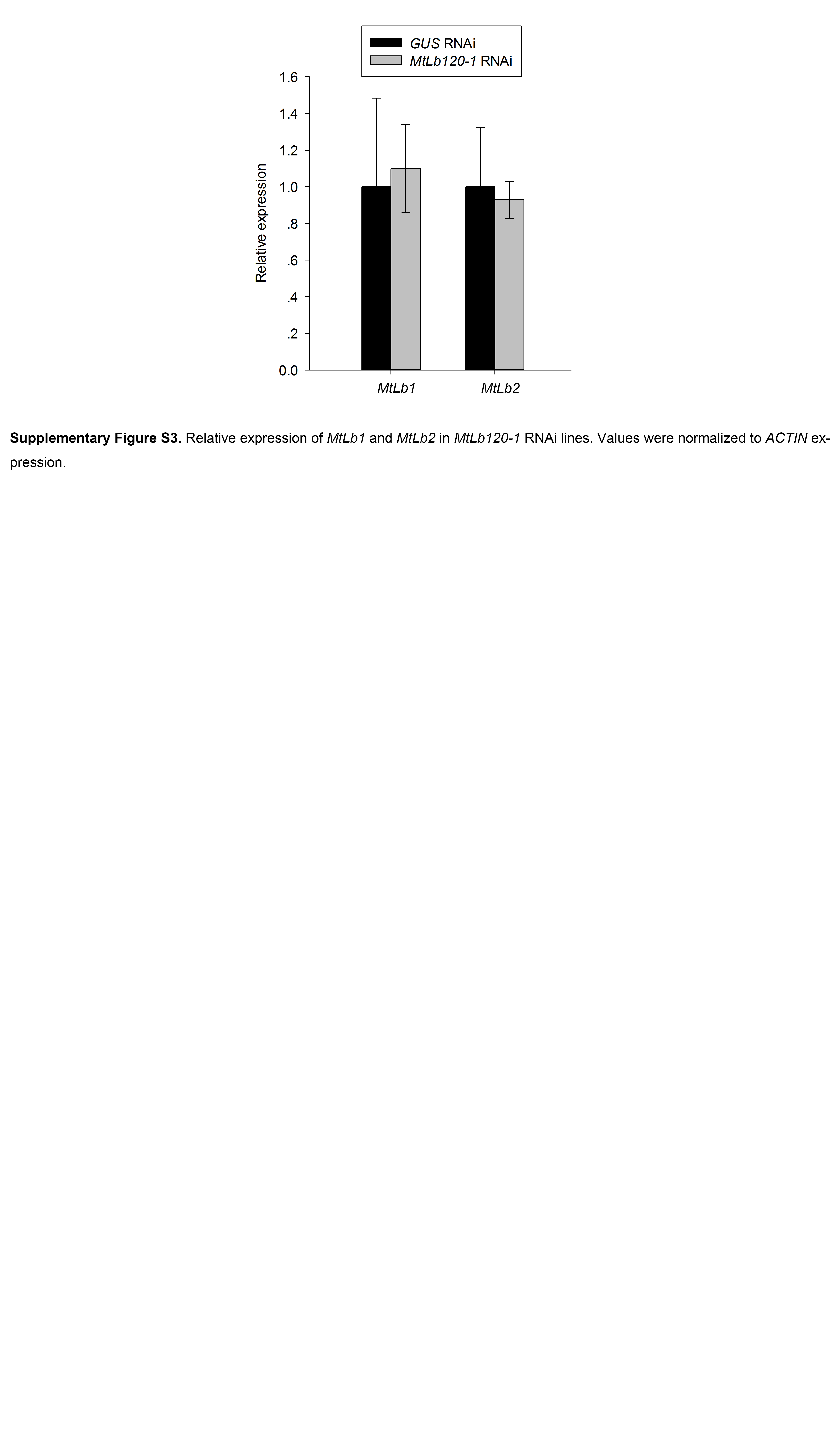

Supplement: Supplementary file 6 [file Image_3.TIF]

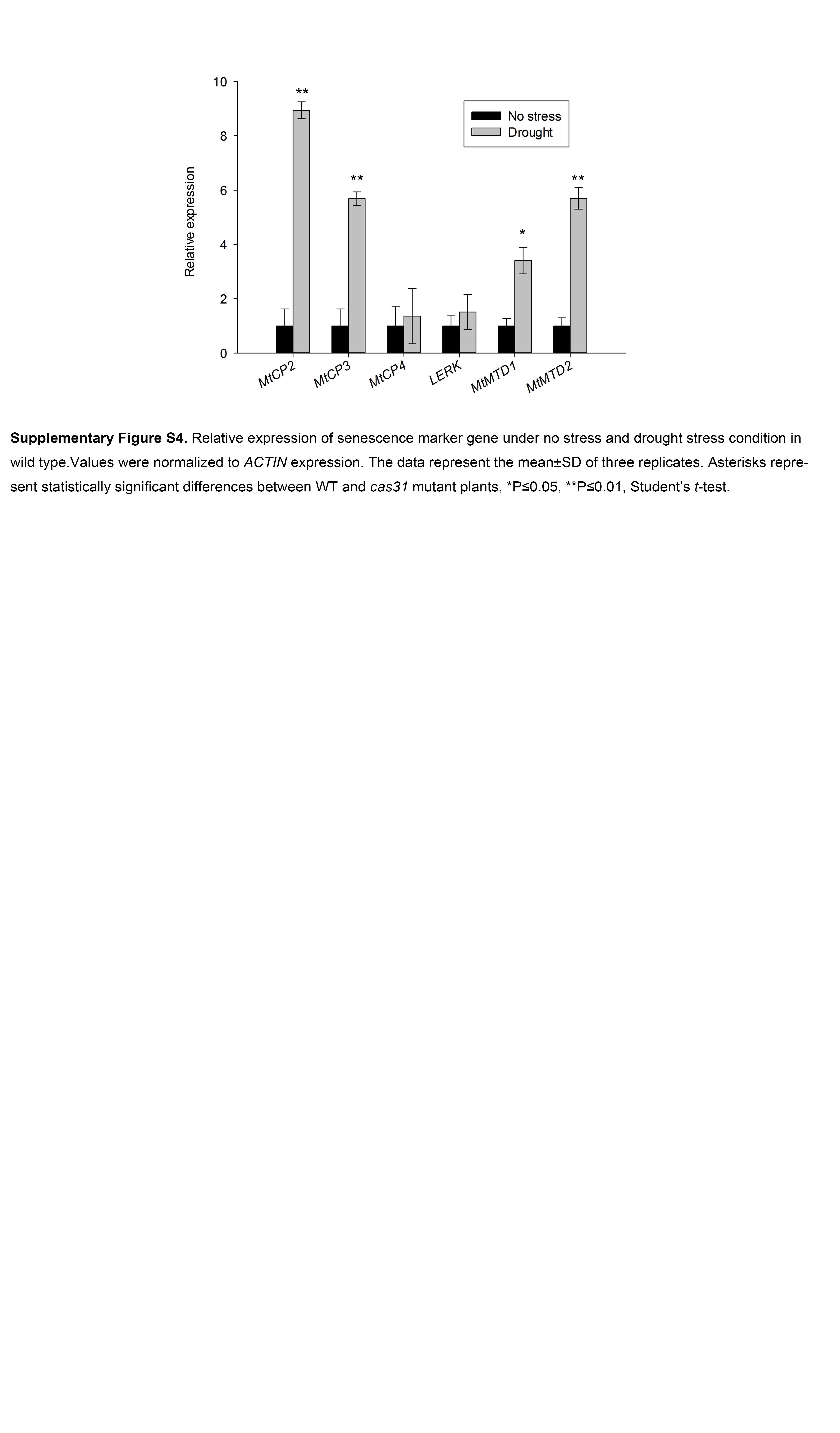

Supplement: Supplementary file 7 [file Image_4.TIF]

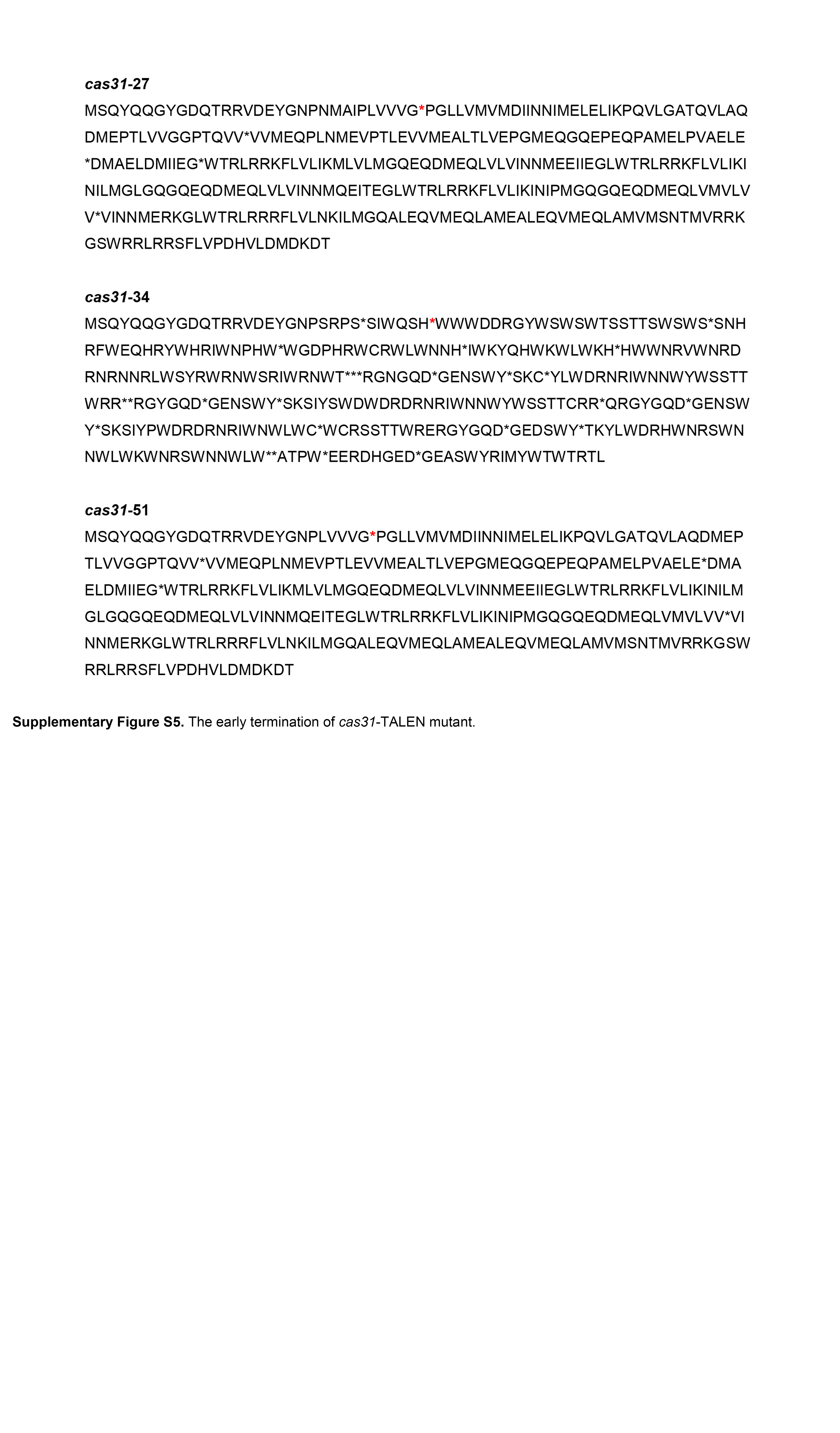

Supplement: Supplementary file 8 [file Image_5.TIF]
